# BMP7 retards peripheral myelination by activating p38 MAPK in Schwann cells

**DOI:** 10.1038/srep31049

**Published:** 2016-08-05

**Authors:** Xiaoyu Liu, Yahong Zhao, Su Peng, Shuqiang Zhang, Meihong Wang, Yeyue Chen, Shan Zhang, Yumin Yang, Cheng Sun

**Affiliations:** 1 Jiangsu Key Laboratory of Neuroregeneration, Nantong University, 19 Qixiu Road, Nantong, Jiangsu 226001, P.R. China; 2 Co-innovation Center of Neuroregeneration, Nantong University, Nantong, Jiangsu 226001, P.R. China; 3 School of Medicine, Nantong University, 19 Qixiu Road, Nantong, Jiangsu 226001, China

## Abstract

Schwann cell (SC) myelination is pivotal for the proper physiological functioning of the nervous system, but the underlying molecular mechanism remains less well understood. Here, we showed that the expression of bone morphogenetic protein 7 (BMP7) inversely correlates with myelin gene expression during peripheral myelination, which suggests that BMP7 is likely a negative regulator for myelin gene expression. Our experiments further showed that the application of BMP7 attenuates the cAMP induced myelin gene expression in SCs. Downstream pathway analysis suggested that both p38 MAPK and SMAD are activated by exogenous BMP7 in SCs. The pharmacological intervention and gene silence studies revealed that p38 MAPK, not SMAD, is responsible for BMP7-mediated suppression of myelin gene expression. In addition, c-Jun, a potential negative regulator for peripheral myelination, was up-regulated by BMP7. *In vivo* experiments showed that BMP7 treatment greatly impaired peripheral myelination in newborn rats. Together, our results established that BMP7 is a negative regulator for peripheral myelin gene expression and that p38 MAPK/c-Jun axis might be the main downstream target of BMP7 in this process.

Myelination of axons is an essential process for the proper physiological functioning of the nervous system, as myelin sheaths allow fast propagation of nerve impulses by saltatory conduction in axons^1^. Defective myelination frequently leads to devastating diseases^2^. Myelin sheaths in the central nerve systems (CNS) and peripheral nerve systems (PNS) are primarily made of oligodendrocytes and Schwann cells (SCs), respectively. SCs are also required for producing extracellular matrix, modulating synaptic activity, supporting nerve development and regeneration, and providing neurotrophic support^3^. It is commonly accepted that transcriptional control is one main regulatory mechanism for the myelination process^4^. Several transcriptional components controlling myelination and differentiation of SCs have been identified, including transcriptional factors Sox10 (SRY-related HMG-box-10), Oct6 (octamer-binding transcription factor-6) and Krox20/Egr2 (early growth response-2)^4^. Sox10 activates Oct6, which synergistically induces the expression of Krox20^5^. Thereafter, Krox20 takes center stage by activating numerous myelin genes such as PMP22 (peripheral myelin protein-22), MPZ (myelin protein zero) and MBP (myelin basic protein). Meanwhile, Krox20 suppresses myelination inhibitors and thus maintains SCs at myelinated state^6^. It has been demonstrated that cyclic AMP (cAMP) signaling pathway is essential for SC myelination *in vivo* and *in vitro*^7^,^8^. Protein kinase A (PKA) is a main downstream effector of cAMP and it plays a pivotal role for inducing myelination in SCs^9^. Recently, one report showed that, at the onset of myelination, G protein-coupled receptor (GPCR) Gpr126 and PKA function as a switch that allows SCs to initiate Krox20 expression and myelination^10^.

The bone morphogenetic proteins (BMPs) belong to the transforming growth factor-β superfamily and were considered as the primary growth factors for inducing the formation of both cartilage and bone^11^. In the canonical pathway, BMPs activate the transcription factor SMAD (Sma and Mad related proteins) by binding to type I and type II serine/threonine kinase receptors^12^. The activated type I BMP receptor phosphorylates the receptor-regulated SMADs (SMAD1/5/8), which triggers the physical interaction between SMAD1/5/8 and SMAD4. Together with SMAD4, SMAD1/5/8 translocate into nucleus for inducing down-stream target gene expression. In addition, the activated receptor complex activates non-SMAD signaling pathway, such as mitogen activated protein kinase (MAPK), PP2A/p70 S6K, RhoA and TAK1/MEKK1^13^. Besides its roles in bone formation, BMPs also play important roles for the development and differentiation of the nervous systems^14^. It has been reported that BMPs can inhibit neural differentiation in embryonic stem cells^15^; conversely, during later neuronal differentiation, BMPs in fact promote differentiation in neuronal cells^16^,^17^. Signaling by BMP4 was shown to block oligodendrocyte precursor cell maturation and regulate the timing of myelination^18^. Recently, it has been demonstrated that BMP4 negatively regulates myelination process in the CNS by affecting the growth and differentiation oligodendrocytes^19^. Sip1 has been identified as an essential modulator of CNS myelination by antagonizing BMP receptor-activated SMAD activity^20^. However, till to date, there is no report concerning BMPs and PNS myelination.

In the present work, we showed that BMP7 significantly attenuates cAMP-induced myelin gene expression by activating p38 MAPK in SCs. Moreover, the application of BMP7 also impairs peripheral myelination in newborn rats. These results showed that BMP7 is a negative regulator for peripheral myelination and is potentially a drug target for the treatment of disorders associated with dysregulated peripheral myelination, such as the Charcot-Marie-Tooth disease.

## Results

### BMP and myelin gene expression profiles during peripheral myelination

**Figure 1:**
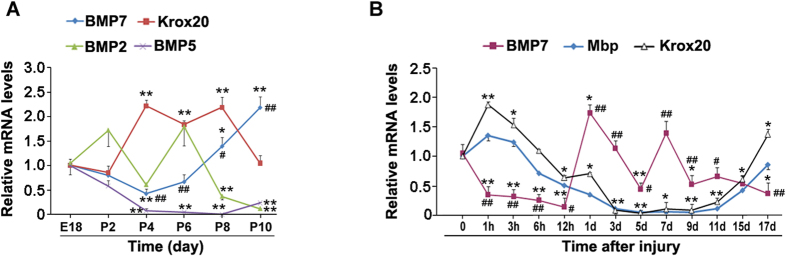
BMP and myelin gene expression profiles during myelination. (**A**) The sciatic nerves from embryonic day 18 (E18) to postnatal 10 days (P10) rats were collected. BMP2, BMP5, BMP7 and Krox20 mRNA levels were analyzed by q-PCR. (**B**) Adult rat sciatic nerves were subjected to crush injury and the injured sciatic nerves were collected at different time points as indicated after injury. BMP7, Mbp and Krox20 mRNA levels were analyzed by q-PCR. n = 4 for each group. Error bars are ± SEM. *p < 0.05, **p < 0.01 versus E18 (A) or time 0 (B); ^#^p < 0.05, ^##^p < 0.01 versus Krox20 at the same time point. Student’s *t* test.

To determine whether BMPs are involved in the PNS myelination process, we first measured the mRNA levels of BMPs and Krox20 in sciatic nerves during development. It has been shown that MBP and MPZ gene expressions were peaked at 6 d post-birth^21^. The protein level of MPZ was detected at sciatic nerves after birth and it continuously increased until 3-week of age^22^. The PNS myelination in rats starts after birth and the first postnatal week is an extremely important period for proper development of PNS myelination^23^. We therefore measured BMP2-7 expression profiles in the sciatic nerves isolated from rats at different ages from embryonic day 18 (E18) to postnatal 10 days (P10). Krox20 mRNA levels are quite low at the beginning, start to increase after P2, and maintain such high expression until P8 (Fig. 1A). The mRNA levels of BMPs (BMP3, BMP4 and BMP6) are too low to be measured (data not shown). The mRNA levels of BMP5 was continuously decreased after birth. BMP2 expression profile is in a random pattern. Notably, BMP7 expression is inversely correlated with Krox20. Since Krox20 is a pivotal transcriptional factor for the PNS myelination, we speculate that BMP7 is likely a negative factor for the PNS myelination. To ascertain this notion, we next investigated BMP7 expression in the injured sciatic nerves. After crush injury, sciatic nerves undergo a reconstitution program for re-myelination. BMP7 mRNA levels immediately decrease upon injury and this low expression maintains until 12 h post injury. After that, BMP7 expression was increased markedly and peaked at 1d; and then it was continuously decreased till 5 d (Fig. 1B). As for the myelin genes, the mRNA levels of Mbp and Krox20 were inversely correlated with that of BMP7 (Fig. 1B). These data clearly demonstrate that the expression of BMP7 is inversely correlated with the PNS myelination process regardless whether it is in the normal development or in re-myelination after injury.

### BMP7 attenuates myelin gene expression in SCs

**Figure 2:**
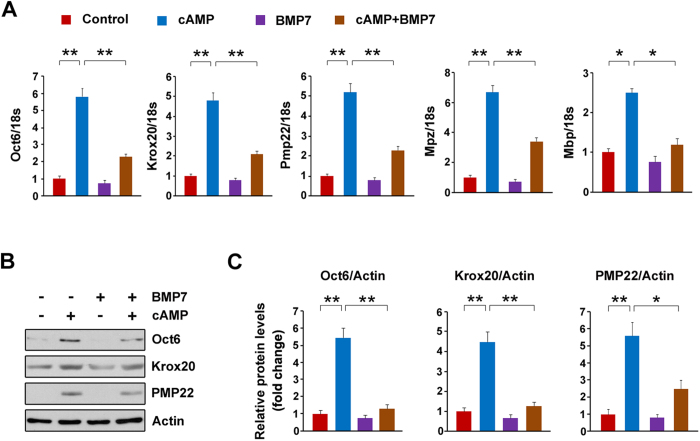
BMP7 attenuates myelin gene expression in the primary rat SCs. The primary rat SCs were treated with BMP7 (50 ng/ml) and cAMP (1 mM) as indicated for 24 h. (**A**) The mRNA levels of Oct6, Krox20, Pmp22, Mpz and Mbp were analyzed by q-PCR. (**B**) The protein levels of Krox20, Oct6 and PMP22 in the SCs were analyzed by Western blot. (**C**) Densitometric quantification of the immunoblot data in (**B**). Actin was used as the loading control. Values represent the average of three independent experiments. Three samples were employed for q-PCR and Western blot analysis. Error bars are ± SEM. *p < 0.05, **p < 0.01, one-way ANOVA with Bonferroni’s *post hoc* testing.

The inverse correlation between the expressions of BMP7 and myelin genes suggests BMP7 is likely a negative regulator of myelination. To test this hypothesis, we treated the cultured primary rat SCs with BMP7 in the presence and absence of cAMP. As shown in Fig. 2A, the myelin genes such as Krox20, Oct6, Pmp22, Mbp and Mpz are all stimulated by cAMP. The application of BMP7 significantly attenuates these stimulations induced by cAMP. Similar to their mRNA levels, the protein levels of Krox20, Oct6 and PMP22 were up-regulated by cAMP, and the application of BMP7 greatly blocked these up-regulations (Fig. 2B,C). These results strongly suggest that BMP7 attenuates myelin gene expression in SCs.

### BMP7 activates p38 MAPK and SMAD in SCs

**Figure 3:**
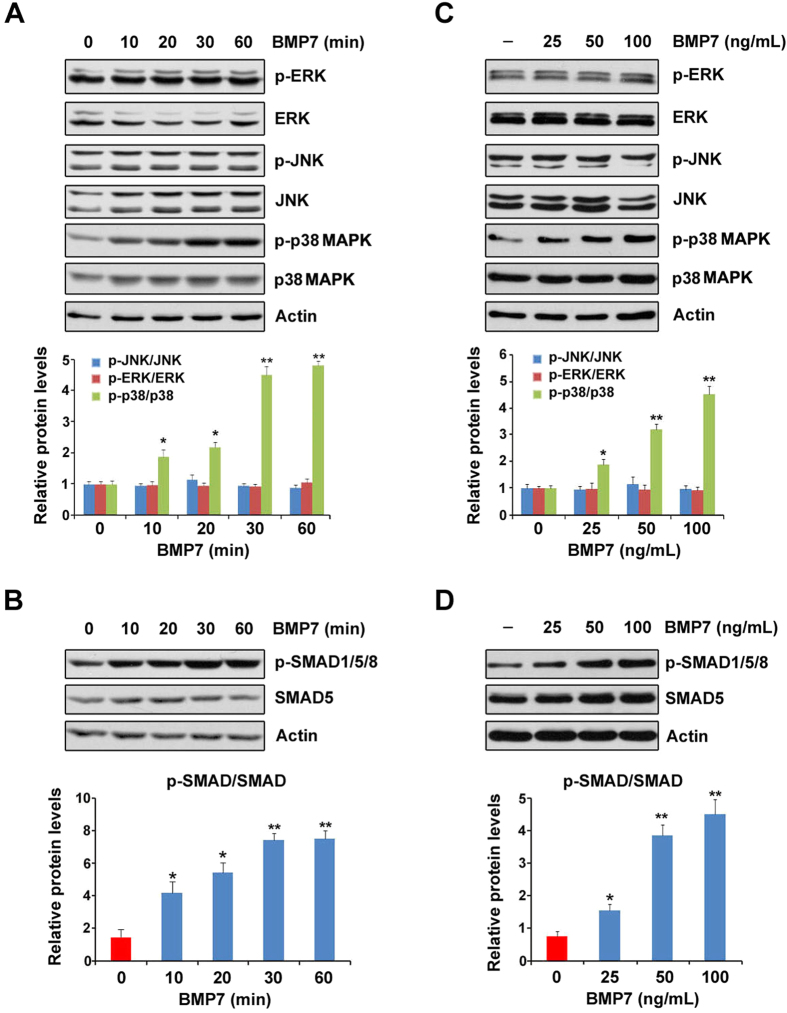
The analysis of BMP7 downstream signal pathways in the primary rat SCs. (**A**,**B**) The cultured primary rat SCs were treated with BMP7 (50 ng/ml) with different time courses as indicated. MAPK (**A**) and SMAD (**B**) pathways were analyzed by Western blot. (**C,D**) The cultured primary rat SCs were treated with BMP7 at different concentrations as indicated for 30 min. MAPK (**C**) and SMAD (**D**) pathways were analyzed by Western blot. Actin was used as the loading control. Data are representative of three independent experiments. Three samples were employed for Western blot analysis. Error bars are ± SEM. *p < 0.05, **p < 0.01, Student’s *t* test.

To determine which signal pathway is responsible for BMP7-mediated suppression of myelin gene expression, we measured the effects of BMP7 on MAPK (mitogen-activated protein kinase) and SMAD pathways in SCs. These two pathways are considered as the main downstream targets of BMPs^13^. The SCs were treated with BMP7 with different exposure duration, and three main effectors in MAPK family were analyzed. The protein levels of phospho-ERK (p-ERK) and phospho-JNK (p-JNK) were not affected by the application of BMP7, while the protein levels of phospho-p38 MAPK (p-p38 MAPK) started to increase after 10 min-incubation of BMP7 and peaked at 30 min (Fig. 3A). The SMAD pathway was continuously activated by BMP7 from 10 to 60 min as evidenced by the constitutive stimulation in phospho-SMAD1/5/8 (p-SMAD1/5/8) protein levels (Fig. 3B). We next examined the dosage effects of BMP7 on these two pathways. As shown in Fig. 3C, p-p38 MAPK was activated by BMP7 in a dose-dependent manner, while p-ERK and p-JNK were not affected. Similar to p-p38 MAPK, p-SMAD1/5/8 was gradually increased by the increasing concentrations of BMP7 (Fig. 3D). These data clearly show that BMP7 treatment activates p38 MAPK and SMAD pathways in SCs.

### The SMAD pathway is not required for BMP7-mediated suppression on myelin gene expression

**Figure 4:**
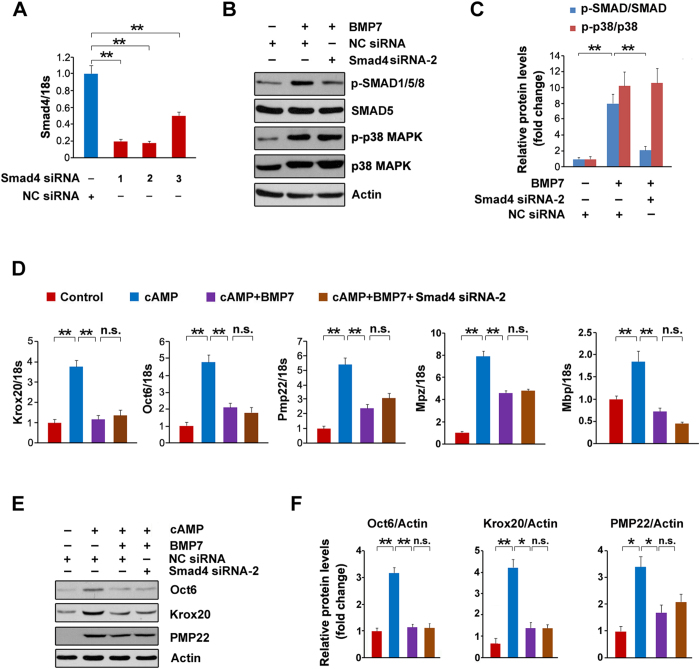
SMAD pathway was not responsible for the inhibitory effects of BMP7 on myelin gene expression. (**A**) The SCs were transfected with SMAD4 siRNAs for 72 h. The mRNA levels of SMAD4 were analyzed by q-PCR. The control cells were transfected with negative control (NC) siRNA. (**B**) The SCs were transfected with SMAD4 siRNA-2 or NC siRNA for 48 h, and then the cells were treated with BMP7 (50 ng/ml) for additional 24 h. The total cell lysates were prepared for measuring SMAD and p38 MAPK by Western blot. (**C**) Densitometric quantification of the immunoblot data in (**B**). (**D,E**) The SCs were transfected with SMAD4 siRNA-2 or NC siRNA. 48 h post-transfection, the cells were treated with BMP7 (50 ng/ml) and cAMP (1 mM) as indicated for additional 24 h. The mRNA levels of Krox20, Oct6 and Pmp22 were analyzed by q-PCR (**D**). The protein levels of Oct6, Krox20 and PMP22 were analyzed by Western blot (**E**). (**F**) Densitometric quantification of the immunoblot data in (**E**). Actin was used as the loading control. Data are representative of three independent experiments. Three samples were employed for q-PCR and Western blot analysis. Error bars are ± SEM. n.s. means no significance. *p < 0.05, **p < 0.01, Student’s *t* test for (**A**); one-way ANOVA with Bonferroni’s *post hoc* testing for (**C**,**D**,**F**).

The above data indicate that BMP7 activates the SMAD pathway, we next wish to explore whether the SMAD pathway is required for BMP7-mediated suppression on myelin gene expression in SCs. SMAD4 is a key coactivator for transducing BMP signaling^13^, thus down-regulation of SMAD4 will antagonize BMP7 evoked SMAD-dependent pathway. For knockdown SMAD4, the SCs were transfected with three pairs of siRNAs against Smad4. The mRNA levels of SMAD4 were dramatically decreased by the siRNAs (Fig. 4A). Of these siRNAs, Smad4 siRNA-2 exhibits the best knockdown efficiency and thus it was chosen for the subsequent experiments. p-SMAD1/5/8 was stimulated by BMP7 while Smad4 knockdown greatly blocks this stimulation (Fig. 4B,C). Meanwhile, we also measured p38 MAPK and the results showed that it was not altered by Smad4 siRNA-2 (Fig. 4B,C). These data indicate that siRNA mediated Smad4 knockdown is a successful strategy for blocking SMAD pathway induced by BMP7, while it has no effect on p38 MAPK. Next we evaluated whether Smad4 knockdown affects myelin gene expression. As shown in Fig. 4D, the expressions of Oct6, Krox20, Pmp22, Mpz and Mbp were stimulated by cAMP, which were greatly blocked by BMP7. Smad4 knockdown fails to restore the BMP7-mediated suppression on myelin gene expression (Fig. 4D). Similarly, the decreases in Oct6, Krox20 and PMP22 protein levels induced by BMP7 were not altered by Smad4 knockdown (Fig. 4E,F). Together, these data indicate that the SMAD pathway is not responsible of the BMP7-mediated suppression on myelin gene expression in SCs.

### p38 MAPK plays a major role for the inhibitory effects of BMP7 on myelin gene expression

**Figure 5:**
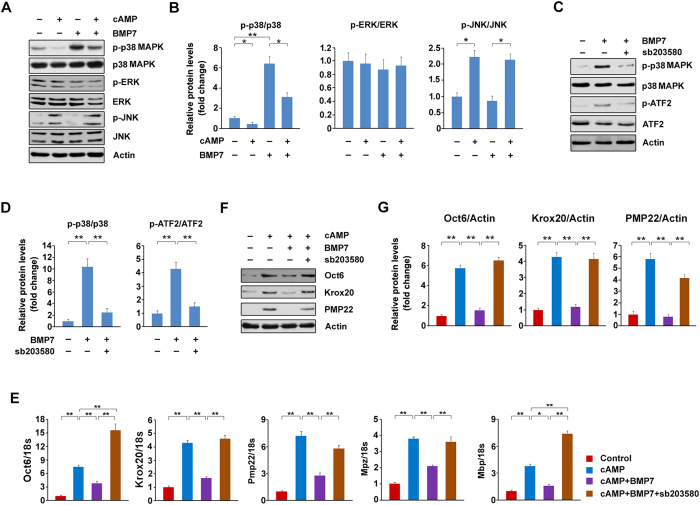
p38 MAPK activation is required for the inhibitory effects of BMP7 on myelin gene expression. (**A**) The SCs were treated with BMP7 (50 ng/ml) and cAMP (1 mM) as indicated and MAPKs were analyzed by Western blot. (**B**) Densitometric quantification of the immunoblot data in (**A**). (**C**) The SCs were treated with BMP7 (50 ng/ml) and sb203580 (10 μM) as indicated for 24 h. The protein levels of p-p38 MAPK and p-ATF2 were analyzed by Western blot. (**D**) Densitometric quantification of the immunoblot data in (**C**). (**E**) p38 MAPK inhibition by sb203580 restores the decreases in myelin gene expression induced by BMP7. The primary rat SCs treated with BMP7 (50 ng/ml), cAMP (1 mM) and sb203580 (10 μM) as indicated and the mRNA levels of Oct6, Krox20, MBP, MPZ and PMP22 was measured by q-PCR. (**F**) p38 MAPK inhibition by sb203580 diminishes the attenuation effects of BMP7 on Krox20, Oct6 and PMP22 protein levels. The SCs were treated as indicated and the protein levels of Krox20 and Oct6 were analyzed by Western blot. (**G**) Densitometric quantification of the immunoblot data in (**F**). Actin was used as the loading control. Data are representative of three independent experiments. Three samples were employed for q-PCR and Western blot analysis. Error bars are ± SEM. n.s. means no significance. *p < 0.05, **p < 0.01, one-way ANOVA with Bonferroni’s *post hoc* testing.

**Figure 6:**
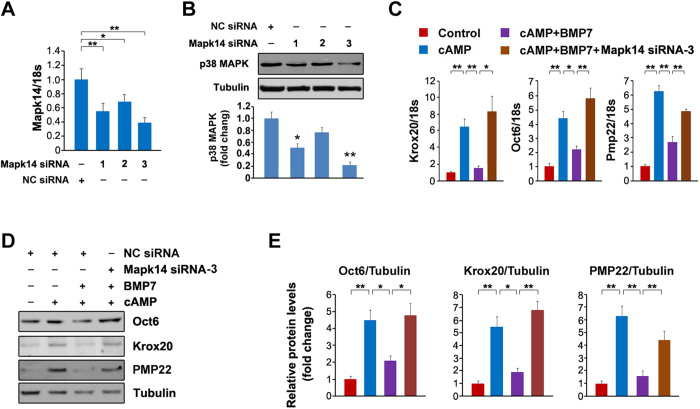
Knockdown of p38 MAPK rescues the blockade effects of BMP7 on myelin gene expression. (**A**,**B**) The primary rat SCs were transfected with Mapk14 siRNAs for 72 h. The mRNA levels of Mapk14 (**A**) or the protein levels of p38 MAPK (**B**) were analyzed by q-PCR and Western blot, respectively. The control cells were transfected with negative control (NC) siRNA. (**C**,**D**) The primary rat SCs were transfected with Mapk14 siRNA-3 or NC siRNA. 48 h post-transfection, the cells were treated with cAMP (1 mM) and BMP7 (50 ng/ml) as indicated. The mRNA levels (**C**) or protein levels (**D**) of Oct6, Krox20 and Pmp22 were measured. Tubulin was used as the loading control. (**E**) Densitometric quantification of the immunoblot data in (**D**). Data are representative of three independent experiments. Three samples were employed for q-PCR and Western blot analysis. Error bars are ± SEM. n.s. means no significance. *p < 0.05, **p < 0.01, Student’s *t* test for (**A**,**B**); one-way ANOVA with Bonferroni’s *post hoc* testing for (**C**,**E**).

Since the SMAD pathway is not involved in the inhibitory effects of BMP7 on myelin gene expression, we next decided to focus on p38 MAPK, another signal pathway activated by BMP7 in SCs. To determine whether p38 MAPK is responsible for BMP7-mediated suppression on myelin gene expression, we treated the SCs with BMP7 in the presence and absence of cAMP and p38 MAPK activity was analyzed. The results showed that p-p38 MAPK was markedly decreased by cAMP, and the application of BMP7 partially restores p-p38 MAPK (Fig. 5A,B). In addition, we also measured ERK and JNK signaling pathways. p-ERK was not affected by cAMP with or without BMP7. As for JNK pathway, p-JNK was stimulated by cAMP and this stimulation was not altered by BMP7 (Fig. 5A,B). Therefore, we speculate that p38 MAPK might be responsible for the BMP7-mediated suppression on myelin gene expression. To test this notion, we first treated the SCs with BMP7 in the presence or absence of sb203580, a potent inhibitor of p38 MAPK. As expected, the application of sb203580 markedly blocks BMP7-induced activation of p38 MAPK (Fig. 5C,D). p-ATF2, a downstream effector of p38 MAPK, was also suppressed by sb203580 (Fig. 5C,D). We next examined whether the inhibitory effects of BMP7 on myelin gene expression is dependent on p38 MAPK. The mRNA levels of Oct6, Krox20, Pmp22, Mbp and Mpz were dramatically increased by cAMP and these increases were largely blocked by BMP7 (Fig. 5E). The application of sb203580 completely restores the BMP7-mediated suppressions on Krox20, Mpz and Pmp22; Oct6 and Mbp were even higher than those observed in the cells treated with cAMP alone (Fig. 5E). In addition, we also checked the protein levels and the results showed that p38 MAPK inhibition by sb203580 could counteract the inhibitory effects of BMP7 on Oct6, Krox20 and PMP22 (Fig. 5F,G). Furthermore, we down-regulated p38 MAPK expression by the siRNAs against Mapk14. As shown in Fig. 6A, the expression of Mapk14 was markedly decreased by the tested three pairs of siRNAs, and Mapk14 siRNA-3 showed the best efficiency. This conclusion was further ascertained by measuring the protein levels of p38 MAPK (Fig. 6B). Similar to the application of sb203580, p38 MAPK knockdown could rescue the BMP7-induced decreases in the mRNA levels of Oct6, Krox20 and Pmp22 (Fig. 6C). The protein levels of Oct6, Krox20 and PMP22 were also rescued by Mapk14 siRNA-3 (Fig. 6D,E). These data strongly indicate that BMP7 attenuates myelin gene expression in SCs by activating p38 MAPK.

### Activation of p38 MAPK counteracts the effects of p38 MAPK inhibition or knockdown on myelin gene expression

**Figure 7:**
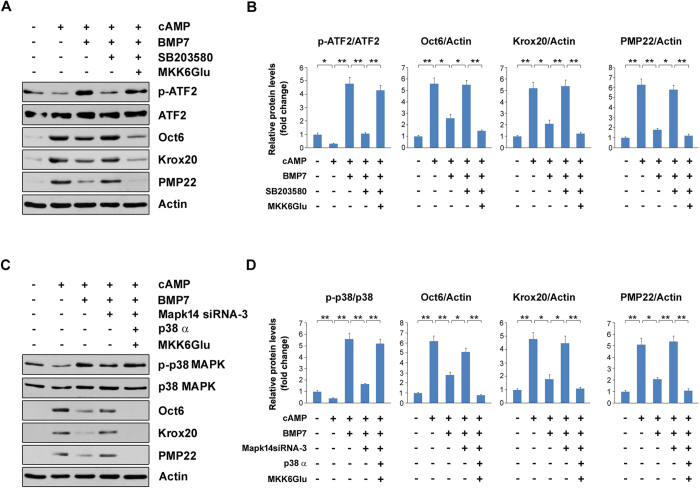
Activation of p38 MAPK counteracts the effects of p38 MAPK inhibition or knockdown on myelin gene expression. (**A**) The SCs were treated with 10 μM sb203580 or vehicle, and then the cells were transfected with plasmid bearing MKK6Glu as indicated. Protein levels were measured by Western blot. Actin was used as a loading control. (**B**) Densitometric quantification of the immunoblot data in (**A**). (**C**) The SCs were transfected with Mapk14 siRNA-3 for 36 h and then the cells were transfected with p38 α and MKK6Glu for additional 12 h. Subsequently, the cells were treated with cAMP and BMP7 as indicated for 24 h. Protein levels were measured by Western blot. Actin was used as a loading control. (**D**) Densitometric quantification of the immunoblot data in (**C**). Error bars are ± SEM. *p < 0.05, **p < 0.01, one-way ANOVA with Bonferroni’s *post hoc* testing.

To further ascertain the pivotal role of p38 MAPK on BMP7-mediated suppressions on myelin gene expression, we re-activated p38 MAPK under p38 MAPK inhibition or knockdown condition in SCs and analyzed myelin gene expression. MKK6 is a up-stream kinase of p38 MAPK and MKK6Glu is a constitutive active form of MKK6. As shown in Fig. 7A, the decrease in p-ATF2 induced by sb203580 was counteracted by the transfection of plasmid bearing MKK6Glu, indicating the inhibition of p38 MAPK by sb203580 was abolished by MKK6Glu. As a result, the rescued myelin gene expression such as Oct6, Krox20 and PMP22 induced by sb203580 were markedly decreased (Fig. 7A,B). Furthermore, we re-activated p38 MAPK under Mapk14 knockdown condition by the co-transfection of plasmids carrying p38α and MKK6Glu. By this regimen, the decrease in p-p38 MAPK induced by Mapk14 siRNA-3 was successfully re-activated (Fig. 7C). As expected, the rescued protein levels of Oct6, Krox20 and PMP22 induced by Mapk14 knockdown were returned to the basal levels (Fig. 7C,D). These data strongly indicate that p38 MAPK plays a major role for mediating the inhibitory effects of BMP7 on myelin gene expression in SCs.

### p38 MAPK/c-Jun axis may accounts for BMP7-mediated suppression on myelin gene expression

**Figure 8:**
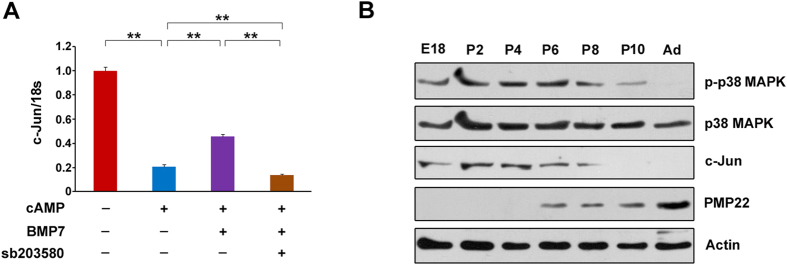
p38 MAPK/c-Jun axis are responsible for the inhibitory effects of BMP7 on myelin gene expression. (**A**) The cultured primary rat SCs were treated as indicated and the c-Jun mRNA levels were detected by q-PCR. (**B**) The protein levels of p38 MAPK, c-Jun and PMP22 in developing rat sciatic nerves isolated from E18, P2, P4, P6, P8, P10 and adult (Ad) rats were analyzed by Western blot. n = 4 for each group. Actin was used as the loading control. Data are representative of three independent experiments. Three samples were employed for q-PCR. Error bars are ± SEM. **p < 0.01, one-way ANOVA with Bonferroni’s *post hoc* testing.

We have shown that the expression of BMP7 is inversely correlated with myelin gene expression during developmental myelination or re-myelination after injury (Fig. 1A,B). Furthermore, we showed that BMP7 attenuates myelin gene expression in SCs by activating p38 MAPK. c-Jun is a potent negative regulator for the PNS myelination^24^. A previous study identified p38 MAPK as a negative modulator for the PNS myelination since p38 MAPK activation is sufficient to up-regulate c-Jun activity^25^. Hence, we speculate that the BMP7-induced retardation of myelin gene expression may result from up-regulation of c-Jun by p38 MAPK. Indeed, we found that c-Jun expression is decreased by cAMP (Fig. 8A), which is consistent with the previous report^24^. BMP7 treatment partially blocks this decrease, and the application of sb203580 could counteract this blockade (Fig. 8A). Furthermore, we measured the protein levels of p-38 MAPK and c-Jun in myelinating sciatic nerves. As shown in Fig. 8B, PMP22 was gradually up-regulated with the time extended, suggesting sciatic nerve myelination was gradually completed. On the other hand, p38 MAPK, as well as c-Jun, continuously decreases after birth. The observed p38 MAPK and c-Jun expression profiles were consistent with the previous reports^24^,^25^. These results indicate that the sciatic nerve myelination process is inversely correlated with p38 MAPK and c-Jun expression. Based up these data, we conclude that the axis of p38 MAPK/c-Jun is likely a main downstream target of BMP7 for its suppression on myelin gene expression.

### Exogenous BMP7 treatment retards peripheral myelination in newborn rats

**Figure 9:**
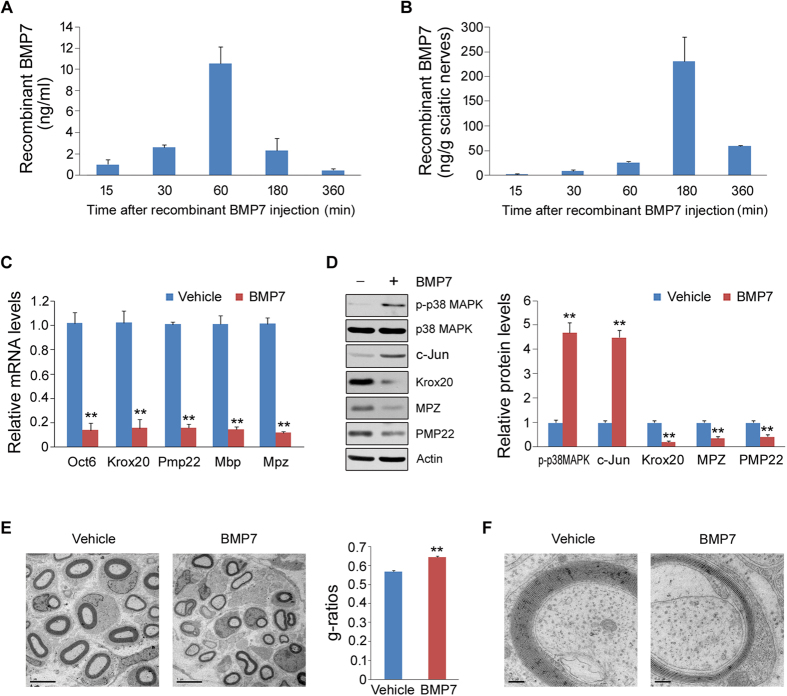
BMP7 treatment retards peripheral myelination in newborn rats. (**A**,**B**) Recombinant BMP7 concentrations in blood (**A**) and sciatic nerves (**B**) after BMP7 injection. (**C**) The mRNA levels of Oct6, Krox20, Pmp22, Mbp and Mpz in sciatic nerves were analyzed by q-PCR. (**D**) The protein levels of p-p38 MAPK, p38 MAPK, Krox20, MPZ and PMP22 were analyzed by Western blot. (**E**,**F**) Analysis of sciatic nerves from vehicle- or BMP7-treated newborn rats by electron microscopy. Scale bar represents 5 μm (**E**) and 0.2 μm (**F**). n = 5 for each group. Error bars are ± SEM. **p < 0.01, Student’s *t* test.

To examine whether BMP7 affects peripheral myelination via down-regulation of myelin gene expression, we treated the newborn rats with recombinant BMP7 at the dosage of 5 ng/g/day for consecutive 10 days. First, we analyzed the recombinant BMP7 concentrations in blood and sciatic nerves. As shown in Fig. 9A, the recombinant BMP7 in blood was detected at 15 min after injection and it peaked at 60 min. In sciatic nerves, the recombinant BMP7 was rather low at 15 min and it increased continuously thereafter and peaked at 180 min (Fig. 9B). Next, we analyzed myelin gene expression and found that the mRNA levels of Oct6, Krox20, Pmp22, Mbp and Mpz were significantly reduced in the BMP7-treated rats (Fig. 9C). Accordingly, the protein levels of Krox20, PMP22 and MPZ were markedly decreased by BMP7 (Fig. 9D). On the contrary, p-p38 MAPK and c-Jun were stimulated by the application of BMP7 (Fig. 9D). Furthermore, we analyzed sciatic nerve morphology by electron microscopy. As shown in Fig. 9E, the thickness of myelin sheath was greatly decreased in the BMP7-treated rats. The statistical analysis for g-ratios also confirmed this notion. The myelin sheath layers were largely reduced by BMP7 treatment (Fig. 9F). These data clearly indicate that BMP7 really retards peripheral myelination *in vivo*.

## Discussion

BMPs are the largest subgroup of the transforming growth factor-β (TGF-β) superfamily of cytokines^26^. They were originally identified as master regulators for inducing ectopic bone formation *in vivo*^27^. Extensive studies have shown that their effects are well beyond the induction of bone formation. To date, over 20 members of the BMP subgroup have been identified. Of these, BMP7, perhaps the best studied BMP subgroup member, functions in bone formation, kidney development and brown fat adipogenesis^28^,^29^,^30^. In the present study, we identified BMP7 as a negative regulator of myelin gene expression via p38 MAPK activation in the cultured primary rat SCs.

The transition of immature SCs to myelinating cells requires pro-myelin gene regulatory proteins, including at least Krox20, Oct6, NFATc4, Brn2 and Sox10^4^. Peripheral myelination in rats is a post-natal event. In newborn rats, fetal nerve fibers comprise several small axons surrounded by a single thin layer of SC cytoplasm. Thereafter, promyelin nerve fibers appear and a one-to-one relationship has been established between each SC and an axon. The SC plasmalemma has spirally enveloped the axon to form myelin sheath. At the age of one week, myelination is well under way, with an average of about 25 compact lamellae/sheath around the myelinated fibers^31^. In this study, we analyzed the expressions of myelin genes and BMP7 in the sciatic nerves from embryonic and newborn rats. An inverse correlation between the mRNA levels of Krox20 and BMP7 was observed during P4 to P10 (Fig. 1A). After injury, SCs undergo dedifferentiation and proliferation. They form Bunger bands at injury sites, resulting in a permissive environment for axon regeneration and remyelination^32^. Once SCs contact the regrowing axons, they start remyelination about 8 days after injury^32^. Our results showed that Krox20 and Mbp was up-regulated at 11 days post-injury (Fig. 1B). In this model, we also noticed an inverse correlation between Krox20 and BMP7 was occurred from 1 h to 11d after injury (Fig. 1B). These data suggest that BMP7 is likely a negative regulator of myelin gene expression. Consistent with our results, signaling by BMPs such as BMP4 was shown to block oligodendrocyte precursor cell maturation and regulate the timing of myelination^18^,^19^,^33^.

In the present study, we show that BMP7 negatively regulates cAMP-induced myelin gene expression in SCs. To determine the underlying molecular mechanisms, we measured two targets of BMP7, MAPKs and SMAD^13^. We found that p38 MAPK and SMAD are both activated by the application of BMP7 in the SCs (Fig. 3A–D). Although the SMAD pathway was previously identified as an important negative regulator for the CNS myelination^20^, our pharmacological intervention experiments excluded the possibility that SMAD was involved in the BMP7-mediated retardation of myelin gene expression (Fig. 4D,E). We subsequently focused our attention on p38 MAPK for addressing the underlying molecular mechanism. p38 MAPK, like JNK and ERK, belongs to MAPKs. These serine/threonine MAPKs relay extracellular signals to the intracellular machinery that regulates a plethora of cellular processes^34^,^35^. In the present study, we observed that BMP7 stimulates p38 MAPK in the cultured SCs (Fig. 3A,C), which is consistent with several previous studies showing that BMP7 activates p38 MAPK in brown pre-adipocyte, metastatic prostate cancer cells and human embryonic stem cells^28^,^36^,^37^. It is worthy to point out that while others reported that JNK activity was activated by BMP7 in mouse neuroblastoma cells and nephrogenic zone derived cells^38^,^39^, we did not observe any noticeable effects of BMP7 on either JNK or ERK in the SCs. The discrepancy is likely due to the different cell lines used for measuring JNK activity, since the dosage of BMP7 and treatment duration are comparable between the present study and the previous studies.

A growing body of evidence suggests that MAPK activities tightly link the myelination processes both in the CNS and the PNS. For example, c-Jun is a downstream target of JNK, which is an important negative regulator for the PNS myelination^24^. Deletion of ERK1/2 in Schwann precursor cells causes disrupted differentiation and marked hypomyelination of axons^40^. Moreover, ERK1/2 also plays a dominant role for promoting rapid myelin growth to increase its thickness, following oligodendrocyte differentiation and initiation of myelination^41^. Similarly, p38 MAPK has been identified as an important regulator for myelination both in the PNS and the CNS. p38 MAPK inhibitors completely and irreversibly block myelination of dorsal root ganglion neurons by oligodendrocytes and prevent the axolemmal organization of the axo-glial adhesion molecule Caspr^42^,^43^. For the PNS myelination, p38 MAPK primarily directs SC differentiation and peripheral myelination by regulating Krox20 expression via its downstream effectors (MK2 and MSK-1/CREB) and transcriptional factors (SCIP and Sox10)^44^. However, a recent report showed that p38 MAPK activation promotes denervated SC phenotype and functions as a negative regulator of SC differentiation and myelination^25^. In our study, we observed that p38 MAPK inhibition reverses the attenuation effect of BMP7 on myelin gene expression in the SCs (Fig. 5E,F). Furthermore, knockdown of p38 MAPK completely counteracts BMP7-induced decreases in myelin gene expression (Fig. 6C,D). Consistent with our results, Yang *et al*. found that enforced p38 MAPK activation blocks cAMP-induced expression of Krox20 and myelin proteins, while inhibition of p38 MAPK in Schwann cell-neuron cocultures promotes myelin formation^25^.

The molecular mechanism responsible for BMP7-mediated inhibition of peripheral myelination is still unclear. One possible explanation is the up-regulation of c-Jun induced by BMP7 in SCs. It has been demonstrated that c-Jun is a negative regulator for SC myelination^24^. In fact, our data did show that cAMP greatly inhibits c-Jun expression and such inhibition was partially restored by the application of BMP7 (Fig. 8A). Ectopic activation of p38 MAPK in the differentiated SCs is sufficient to induce c-Jun expression and promote dedifferentiation^25^. p38 MAPK was gradually decreased during the PNS myelination (from E18 to Ad) and c-Jun follows a similar pattern. One previous report also showed that p38 MAPK in rat sciatic nerves was decreased during peripheral myelination process^25^. On the contrary, PMP22 was rather low until P4 and it was continuously increased thereafter (Fig. 8B). It has been demonstrated that PMP22 protein accumulates between birth and postnatal day 30 and then reaches a plateau^31^, which is in a agreement with our results. Based on these data, we conclude that BMP7 activates p38 MAPK and leads to c-Jun expression, eventually resulting in the retardation of myelin gene expression. Notably, BMP7 expression in sciatic nerves was rather low at the beginning of birth and it increased gradually along with the time extended. However, p-p38 MAPK was high at the beginning of birth and it decreased continuously thereafter. The reason for this inconsistence is likely due to circulated BMP7 which was produced by other tissues.

Taken together, our present study show that BMP7 retards SC myelin gene expression by activating p38 MAPK. BMP7 is a ubiquitous TGF-β cytokine, and elucidating the spatial and temporal expression of BMP7 *in vivo* is a potential strategy for manipulating the PNS myelination process. By showing that BMP7 is a negative regulator for peripheral myelination, our present study provides novel insights into developing therapeutic strategies for treating the PNS neuropathies.

## Methods

### Bioreagents

Recombinant human BMP7 and recombinant human neuregulinβ-1 were obtained from PeproTech (Rocky Hill, NJ, USA). Rabbit anti-PMP22, rabbit anti-Krox20 antibodies, cAMP, DAPI, PMSF, leupeptin, aproptonin, okadaic acid and N^6^,2′-O-Dibutyryladenosine 3′,5′-cyclic monophosphate sodium salt (cAMP) were purchased from Sigma-Aldrich (St. Louis, MO, USA). Polyvinylidene fluoride (PVDF) membrane, chemiluminescence reagents and sb203580 were from Millipore (Billerica, MA, USA). Rabbit anti-phospho-Erk, rabbit anti-Erk, rabbit anti-phospho-JNK, rabbit anti-JNK, rabbit anti-phospho-p38 MAPK, rabbit anti-p38 MAPK, rabbit anti-phospho-SMAD1/5/8, rabbit anti-SMAD5, rabbit anti-phospho-ATF2, rabbit anti-ATF2 and rabbit anti-Tubulin antibodies were purchased from Cell Signaling Technology (Danvers, MA, USA). Mouse anti-Actin, rabbit anti-Oct6, HP-conjugated goat anti-mouse and HP-conjugated goat anti-rabbit antibodies were from Santa Cruz Biotechnology (Dallas, Texas, USA). Rabbit anti-c-Jun and anti-PMP22 antibodies were from Abcam (Cambridge, MA, USA). Fetal bovine serum, DMEM, RNAiMAX, Opti-MEM, Lipofectamine and penicillin-streptomycin were from Life Technologies (Carlsbad, CA, USA). SYBR Green Supermix and cDNA synthesis kit were from Bio-Rad (Hercules, CA, USA). All other chemicals and regents were of analytical grade.

### Sciatic nerve injury model

The procedures for sciatic nerve crush injury model in rats were described previously^45^. Briefly, adult, male Sprague-Dawley (SD) rats (180–200 g) were anaesthetized before the sciatic nerve was exposed through an incision on the mid-thigh of left hind limb. 3-mm long nerve was crushed two times (15 seconds each time, 3 seconds interval) with a hemostatic forceps. A 3-mm long crushed nerves, together with both nerve ends (1 mm long), were harvested at different time points as indicated in Fig. 1B. All of the animal protocols were approved by the Animal Care and Use Committee of Nantong University and the Jiangsu Province Animal Care Ethics Committee. The procedures for sciatic nerve injury model in rats were carried out in accordance the approved guidelines.

### Newborn rat treatments

1 day-old newborn rats (P1) were received recombinant BMP7 via hypodermic injection at the dosage of 5 ng/g body weight/day for consecutive 10 days. After treatment, sciatic nerves were taken for further analysis.

### Schwann cell cultures and treatments

Primary SCs were prepared from sciatic nerves from postnatal day 1 rats^46^. Afterwards, SCs were cultured on polylysine-coated dishes in M medium (DMEM with 10% FBS, 50 μg/ml penicillin-streptomycin) supplemented with 10 nM cytosine arabinoside (AraC) at 37 °C in 95% humidified air/5% CO_2_. After 2 days, the medium were changed and Schwann cells were cultured in DMEM containing 10% FBS, 50 μg/ml penicillin-streptomycin, 2 μM forskolin, and 5 ng/ml of the EGF domain of recombinant human neuregulinβ-1 (HRG), which is called M^+^ medium. For studies on the effects of BMP7 analogues on SC differentiation, SCs were either starved overnight in M^+^ medium prior to treatment with 50 ng/ml BMP7 for 24 h. To stimulate myelin gene expression, cells were treated with 1 mM cAMP. To inhibit p38 MAPK, cells were treated with 10 μM sb203580. To stimulate p38 MAPK, cells were transfected with MKK6Glu plasmid (Addgene, plasmid #13518) alone or together with p38α plasmid (Addgene, plasmid #20351) by using Lipofectamine.

### Recombinant BMP7 assay

To examine the pharmacokinetics of BMP7 *in vivo*, we administered the newborn rats with the recombinant human BMP7 at the dosage of 5 ng/g body weight via hypodemic injection. Blood and sciatic nerves were collected at different time points after injection. Recombinant human BMP7 concentrations in blood and sciatic nerves were analyzed by a human BMP7 ELISA kit (Cusabio, Wuhan, China) according to the manufacturer’s instructions.

### Transmission electron microscopy

The sections of sciatic nerves were fixed in pre-cooled 2.5% glutaraldehyde for 3 h and 1% osmium tetraoxide solution for 1 h. After washing and dehydration, the sections were embedded in Epon 812 epoxy resin and cut into-thin sections of 60 nm thickness to be stained with lead citrate and uranyl acetate. The stained sections were observed under a transmission electron microscope (JEO Ltd., Tokyo, Japan). Images were taken from 10 random fields to determine the number of myelin sheath layers, the thickness of myelin sheaths and the diameter of myelinated nerve fibers using Image Pro Plus software (Media Cybernetics, Silver, Spring, MD). Mean g-ratios were calculated with a correction including nonmyelinated axons >1 μm diameter.

### Total protein extraction from cells and tissues

Cells were lysed in the lysis buffer (25 mM Tris-HCl, pH 7.4; 10 mM NaF; 10 mM Na_4_P_2_O_7_; 2 mM Na_3_VO_4_; 1 mM EGTA; 1 mM EDTA; 1% NP-40; 10 μg/ml leupeptin; 10 μg/ml aproptonin; 1 mM PMSF and 20 nM okadaic acid). After 20-min rotation at 4 °C, cell lysates were centrifuged at 13,200 rpm for 20 min at 4 °C. Tissues were homogenized with a dounce homogenizer in the ice-cold tissue lysis buffer (25 mM Tris-HCl, pH 7.4; 100 mM NaF; 50 mM Na_4_P_2_O_7_; 10 mM Na_3_VO_4_; 10 mM EGTA; 10 mM EDTA; 1% NP-40; 10 μg/ml leupeptin; 10 μg/ml aproptonin; 2 mM PMSF and 20 nM okadaic acid). After homogenization, lysates were rotated for 1 h at 4 °C and then centrifugated at 13,200 rpm for 20 min at 4 °C. Supernatants were collected and protein concentration was quantified by using Protein Assay Kit (Bio-Rad). The concentrations of protein were normalized with lysis buffer to have equivalent amounts of protein and volume. Protein was denatured by boiling at 100 °C for 5 min in 1 X Laemmli buffer. The lysates were cooled to room temperature before loading for Western blot analysis.

### Knockdown of Smad4 and Mapk14

For gene silence, three pairs of small interference RNAs (siRNAs) against Smad4 or Mapk14 were synthesized (Ribobio CO., LTD, Guangzhou). The siRNAs were transfected into SCs by RNAi MAX (Life technologies) according to the manual instructions. Cells transfected with negative control (NC) siRNA were used as control.

### Western blot analysis

Western blot analysis was performed as previously described^47^. Samples from cell lysates or tissue lysates were resolved by SDS-PAGE and then transferred to polyvinylidene fluoride (PVDF) membrane. After 1 h blocking at room temperature using 10% blocking reagent (Roche), membrane was incubated overnight with primary antibody in Tris-buffered saline solution/Tween (TBST) containing 10% blocking reagent at 4 °C. After the incubation, membrane was washed three times in TBST and incubated with secondary antibody for 1 h at room temperature. After three-time washing in TBST, membrane was developed using a chemiluminescence assay system (Roche) and exposed to Kodak exposure films. Densitometric quantification of the immunoblot data was performed by using the software of Quantity-One (Bio-Rad).

### Quantitative real-time PCR

Total RNA was extracted from animal tissues using Trizol reagent and transcribed into cDNA using cDNA synthesis kit. The gene expression analysis was performed with StepOne Real-Time PCR Detection System (Applied Biosystems) with SYBR Green Supermix. The mRNA level was normalized to 18S as a house keeping gene. The primer sequences used were: 18S rRNA forward: 5′-AGTCCCTGCCCTTTGTACACA-3′; 18S rRNA reverse: 5′-CGTTCCGAGGGCCTCACT-3′; *Mbp* forward: 5′-GGC ATC ACA GAA GAG ACC CTC AC-3′; *Mbp* reverse: 5′-GCC CGA TGG AGT CAA GGA TG-3′; *Mpz* forward: 5′-GGA GGC CGA GAT GCC ATT TC-3′; *Mpz* reverse: 5′-TGC CGT TGT CAC TGT AGT CTA GGT T-3′; *Oct6* forward: 5′-TGG GCC TAG CGC ACC CTC AAT G-3′; *Oct6* reverse: 5′-GGT ACT GCC ACC GCC TGC CTT G-3′; *Pmp22* forward: 5′-ATC TCA AAG CCT TCG TCA CTC C-3′; *Pmp22* reverse: 5′-GGC CAA TAC AAG TCA TCG CTA G-3′; *Krox20* forward: 5′-GAT CCT TCA GCA TTC TTA TCG-3′; *Krox20* reverse: 5′-CAG GAT AGT CTG GGA TCA TAG-3′. *Smad4* forward: 5′-CAA CTC TCC AAT GTC CAC AG-3′; *Smad4* reverse: 5′-TCA CGG TCC AGG TAG TAA C-5′; *Mapk14* forward: 5′-ACA CAG CCA AGT CGT CAA-3′; *Mapk14* reverse: 5′-CCA TCA GAA GGA ACC ACA CT-3′.

### Statistics and data analyses

Data are presented as a mean ± SEM in the graphs. Quantifications were performed at least three independent experimental groups. The comparisons between two groups were performed using unpaired two-tailed Student’s *t*-test. For multiple-group comparisons, one-way ANOVA with Bonferroni’s *post hoc* test was applied to evaluate for no differences among the group means. p < 0.05 was considered statistically significant.

## Additional Information

**How to cite this article**: Liu, X. *et al*. BMP7 retards peripheral myelination by activating p38 MAPK in Schwann cells. *Sci. Rep.* **6**, 31049; doi: 10.1038/srep31049 (2016).
